# Assessing the Acceptability and Usability of an Internet-Based Intelligent Health Assistant Developed for Use among Turkish Migrants: Results of a Study Conducted in Bremen, Germany

**DOI:** 10.3390/ijerph121214987

**Published:** 2015-12-03

**Authors:** Florence Samkange-Zeeb, Sinja Alexandra Ernst, Funda Klein-Ellinghaus, Tilman Brand, Anna Reeske-Behrens, Till Plumbaum, Hajo Zeeb

**Affiliations:** 1 Leibniz Institute for Prevention Research and Epidemiology—BIPS, Achterstraße 30, 28359 Bremen, Germany; samkange@bips.uni-bremen.de (F.S.-Z.); ernst@bips.uni-bremen.de (S.A.E.); kleinelf@bips.uni-bremen.de (F.K.-E.); brand@bips.uni-bremen.de (T.B.); 2 Department of Anthropology and Cultural Research, University of Bremen, Enrique-Schmidt-Str. 7, 28359 Bremen, Germany; 3 Federal Institute for Occupational Safety and Health (BAuA), Friedrich-Henkel-Weg 1-25, 44149 Dortmund, Germany; reeske-behrens.anna@baua.bund.de; 4 Distributed Artificial Intelligence Laboratory, Technical University Berlin, Ernst-Reuter-Platz 7, 10587 Berlin, Germany; till.plumbaum@dai-labor.de

**Keywords:** internet, migrant health, information, technology, prevention

## Abstract

The Internet offers a new chance for health professionals to reach population groups not usually reached through traditional information channels, for example, migrants. Criticism has, however, been raised that most health information on the Internet is not easy to read and lacks cultural sensitivity. We developed an Internet-based bilingual health assistant especially for Turkish migrants in Germany, tested its acceptance, and evaluated its usability in a participatory research design with families with and without Turkish migrant background. The interactive health assistant covered the following: nutrition, physical activity, overweight, diabetes, as well as pregnancy and pregnancy support. The idea of an Internet-based health assistant was generally accepted by all participants of the evaluation study, as long as it would be incorporated in existing appliances, such as smartphones. The bilingual nature of the assistant was welcomed especially by first generation migrants, but migrant participants also indicated that not all health information needed to be made available in a culture-specific way. The participants were least satisfied with the nutrition component, which they felt should include recipes and ingredients from the culture of origin, as well as specific aspects of food preparation.

## 1. Introduction

Modern communication and information technologies are now an established part of daily life and constant developments in their use are being made [1,2,3]. This also affects all aspects of modern health communication. Short-text-messages, applications on smartphones and the Internet are increasingly being used to disseminate health information and reach patients, respectively, the public [4,5,6]. Germany has one of the highest Internet penetrations, covering more than 80% of the country’s population [1]. The proportion of persons reporting to use a smartphone went up from 25% in 2013 to 50% in 2014 [7]. This opens a new chance for health professionals to reach population groups not usually reached through traditional information channels such as those relying on written material. One such population group is that of migrants. Research from different countries, including Germany, has shown that, compared to the majority population, migrants generally have less access to health services and health information, mainly due to language and/or cultural barriers [8,9,10,11,12]. The use of electronic devices in health communication and information, such as mobile phone technologies, is being propagated as means to overcome these barriers as they offer the chance to provide individualized health information [13,14].

Criticism has, however, been raised that most health information on the Internet is not easy to read [15,16] and lacks cultural sensitivity [15]. In Germany, there is a general lack of research studies on the use and acceptability of Internet-based health information. Studies focusing on the development, usability, and acceptability of bilingual and culturally sensitive Internet sites are even scarcer. Within the context of the scientific and technology cooperation between Germany and Turkey, we developed a bilingual Internet-based health assistant for Turkish migrants in Germany and evaluated its acceptance and usability. The aim of the health assistant was to provide information and advice on the following health topics: nutrition, physical activity, overweight, diabetes, as well as pregnancy and pregnancy support.

## 2. Methods

**Figure 1:**
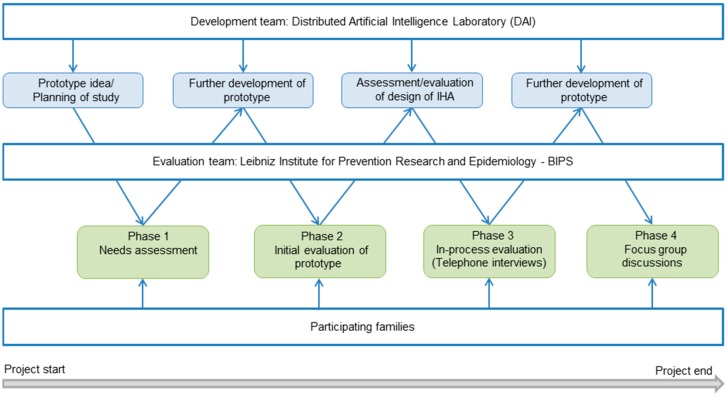
Development and evaluation process of the multilingual intelligent health assistant (IHA).

A qualitative participatory research approach was adopted for the development and evaluation of the intelligent health assistant (IHA). The process was subdivided into four phases, namely: needs assessment, initial evaluation of prototype, in-process evaluation, and a closing focus group discussion (Figure 1).

All study participants were residents of Bremen City, Germany. Turkish migrant background was defined as having one or more of the following characteristics: (a) born in Turkey (b) Turkish nationality; or (c) at least one parent born in Turkey.

**Figure 2:**
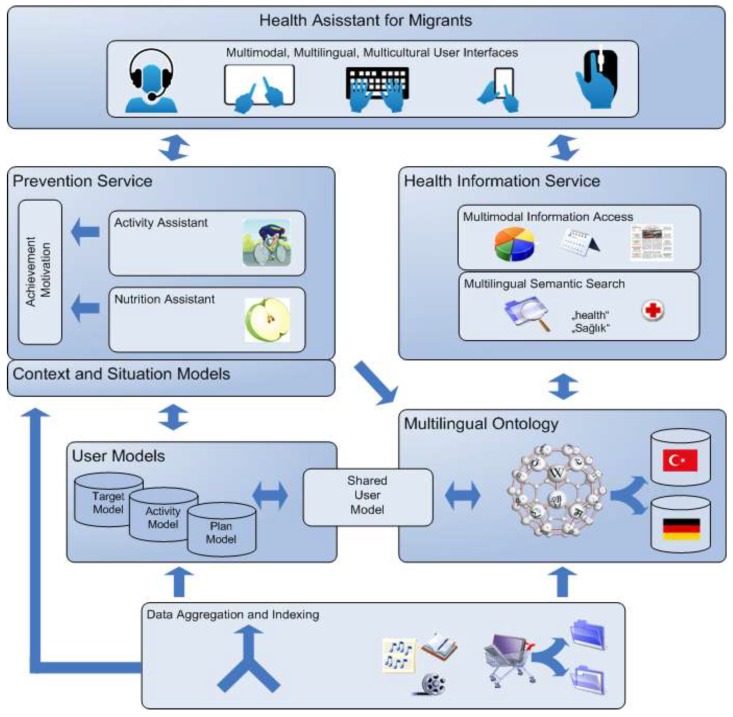
Overview of the system architecture of the IHA.

During the initial phase, families with and without Turkish migrant background were recruited through key persons at family and youth centers, as well as through community networking in the city of Bremen, Germany. Those without Turkish migrant background were also recruited so that they could serve as a comparison group, particularly for the initial needs assessment phase of the study. During this phase, family members participated in qualitative interviews where they were asked about their preferred information sources for health related issues, whether they would use an IHA, what they thought an IHA should look like, what its surroundings should look like and what it should be able to do. The participants’ feedback and ideas were used to further develop the idea of a basic prototype of the health assistant which had been designed by the Distributed Artificial Intelligence Laboratory (DAI) at the Technical University Berlin at the outset of the study. The basic prototype had two main features: a module on prevention and a tailored health information service, with focus on nutrition, physical activity, overweight, diabetes, as well as pregnancy support (Figure 2). The features also included a calendar service, which was set up as the start screen of the prevention service. Participants could use the calendar to note important appointments such as medical check-ups or immunization dates. To ensure a high quality of the information provided, the contents were developed in close collaboration with experts from different disciplines, such as obstetricians and nutritionists. A detailed description of the IHA is presented in the results section.

**Figure 3:**
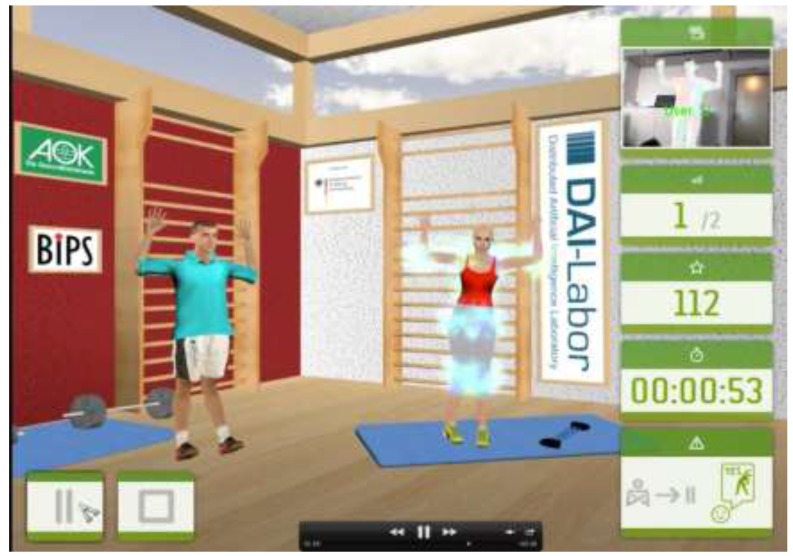
User interface of the Activity Assistant.

In the second phase, participants were contacted and the contents of the up to then developed prototype demonstrated to them. The demonstration took place at the participants’ homes and lasted up to four hours. They were asked to assess the usability of the functions and whether they would use them in their everyday lives (acceptability). Usability was defined and assessed as the ease with which participants could use the respective functions to get the information they desired. This also included the satisfaction of the participants with the presentation form of the information, *i.e.*, use of text or pictures. The participants’ ideas and comments were summarized and forwarded to the IHA developers who incorporated them in the instrument’s further refinement. In the third phase, telephone interviews were conducted with some of the participants who we were able to reach on the phone and were available for the interview. The participants were asked questions concerning the design of the health assistant, e.g., the form and type of the physical activity trainer incorporated in the IHA, and whether they preferred phantasy figures or real people (Figure 3). They were also asked about their preferred background setting of the activity frame: whether abstract, natural environment, or gym. Once again, the participants’ comments and suggestions were reviewed and forwarded to the IHA constructors.

In the last phase of the study, Turkish families participated in a one-day workshop, during which they used the IHA, discussed its acceptability and usability, and made suggestions for further improvement. The IHA guided participants through physical activity exercises. The participants also looked for recipes, practiced using the calendar, and setting reminders for important appointments, such as medical check-ups. They also received a demonstration on how they can use the IHA to store their family’s health data, which is then taken into consideration when they look for recipes or other health information. During the workshop activities, each participant had a member of the evaluation team as a “guide”. The latter sat next to the participants and helped them use the IHA if and where necessary. The participants were encouraged to think loudly during the activities and their thoughts were documented using audio-digital equipment. In addition, the “guides” wrote observational protocols in which they documented the behavior of the participants, including any difficulties they encountered using the IHA, such as problems reading the writing or following instructions.

An important aspect of the study was to develop a culturally-sensitive IHA: an IHA which, in addition to language, would consider similarities and differences of culturally-diverse users in approaches and attitudes towards use of information technology. Hence participants were also explicitly encouraged to reflect on the acceptability and compatibility of the IHA and its features with their own experiences, beliefs, and values and those of their communities at large. At the end of the workshop, a focus group discussion which was facilitated using mind-mapping methods was conducted. All qualitative interviews and the workshop proceedings, as well as the focus group discussions, were audio recorded and transcribed. Turkish interviews were directly translated into German during the listening phase. The acquired data were analyzed based on content analysis methods [17], using the qualitative analysis software MAXQDA. Information from the focus group discussion was extracted from the mind maps and analyzed together with the corresponding audio data. The data were analyzed for differences in expectations, wishes, and ideas between Turkish and German families, different age-groups, and between males and females.

## 3. Results

### 3.1. Features of the IHA

The health assistant was constructed to be interactive, enabling the user to either use it anonymously or personalized. For the latter, the user could enter personal details such as age, sex, weight, as well as relevant (health) details, and then look for tailored information and/or guidance. For example, should an individual suffering from diabetes look for a particular recipe, the offers they receive will take their health condition into consideration. Depending on the user’s preferences, all information could either be shown in one language (German or Turkish) or in a mixture of both languages.

As stated in the methods section, the intelligent health assistant comprised two main services, the health information service and the prevention service. For both services, technologies that provide easy access even for users with little technological experience were selected. These include multilingual features, as well as intuitive and multimodal user interfaces (Figure 2).

#### 3.1.1. Health Information Service

The health information service offered a variety of information related to the topics covered by the health assistant: nutrition, physical activity, overweight, diabetes, as well as pregnancy and pregnancy support. The information included for instance the causes of diabetes, its diagnosis and therapy procedures, as well as prevention measures. This could also be combined, e.g., for diabetes and pregnancy, or pregnancy and physical activity. For the latter the user would get advice on physical activities which can be done during the different phases of pregnancy. The information was offered as a search interface similar to Google that accepted polyglot search queries which could be entered in German or Turkish. The user could, however, also combine both languages and, for instance, enter “*Fettleibigkeit diyetimi nasl ayarlamaliyim?*”, which translated into English is “obesity how should I plan my diet”. The service would then process the query and match the terms to a health ontology. The results could be offerings from health insurances such as nutrition, physical activity and relaxation courses, websites with tips for healthy living, or nearby places offering different activities or services, for example, fitness courses. The websites could be from health insurances or from institutions such as the German Nutrition Society, the German Sports Confederation, or the German Federal Center for Health Education, which all offer some information in different languages including Turkish.

The pregnancy support function could be used to obtain general information on pregnancy as well as health-related topics particularly associated with pregnancy. Furthermore, participants could inform themselves about the whereabouts of local support/offers and classes for pregnant women, e.g., antenatal classes. Additional aspects such as an explanation view showing more details of the search path were also available for interested users. Users could also search for information on other health topics not covered by the health assistant using the health information service.

#### 3.1.2. Prevention Service

The prevention service was made up of two parts: a nutrition assistant and a physical activity assistant. The nutrition assistant could be used by participants to obtain recipes or nutritional values/information tailored to their specific health needs, or those of family members. In addition to providing recipes, the nutrition function could also be used to compile a shopping list for the respective recipe and also provided step-by-step instructions on how to prepare the food.

The physical activity assistant guided participants through a set of recommended exercises. This was done through the use of a virtual trainer in the form of an avatar, which demonstrated the exercises/motions the participants were supposed to do (Figure 3). Similar to the nutrition assistant, participants could enter their personal (health) details and get tailored recommendations. The participants performed the exercises in front of the monitor, while being filmed by a special camera. The performed motions were simultaneously transferred to another virtual avatar, which was immediately shown on the monitor. This allowed for an immediate visual feedback on the participants’ own performance. Should the performance of the participant differ considerably from the demonstrated motions, the trainer avatar gave advice on how the exercises could be improved on. The nutrition and physical activity components were interlinked, thereby enabling the tailoring of participants’ nutritional needs to their activity levels.

### 3.2. Description of Study Participants

Nine Turkish and four German families comprising 28 persons (19 females) aged 11–70 years took part in the first phase of the study (Table 1). A total of 11 persons (three men and eight women) from five families took part in the second phase of the evaluation. All except two had a Turkish migrant background. In phase three, telephone interviews were conducted with three persons (one man and two women) of Turkish migrant background. Eight persons (three men and five women) from four families, all with Turkish migrant background, participated in the focus group discussion workshop.

**Table 1:** Demographic characteristics of persons who participated in the evaluation of the acceptability and usability of the health assistant (first phase of the study).

*N* = 28	No Turkish Migrant Background (*n* = 9) ^1^	Turkish Migrant Background (*n* = 19)
**Age (in years)**		
11–20	4	6
21–30	1	2
31–50	4	8
51–60	-	1
61–70	-	3
**Sex**		
Male	3	6
Female	6	13
**School education**		
University entrance	1	2
Upper secondary school	3	1
Lower secondary school	1	3
College entrance	-	1
Still at school	4	6
No school certificate	-	3
Missing	-	3
**Occupational status**		
Employed	1	4
Maternity leave	2	-
Unemployed	2	9
Still at school	4	6
**Nationality**		
German	8	4
Turkish	-	15
Other	1	-
**Country of birth**		
Germany	8	-
Turkey	-	5
Other	1	14
**Length of stay in Germany (in years)**		
4–10	-	3
11–20	-	3
21–41	1	8
Not applicable	8	5

^1^ Eight persons without any migrant background and one with Brasilian migrant background.

#### 3.2.1. Phase 1: Needs Assessment for Sources of Health Information

Results from the needs assessment indicated that the Internet was the most common preferred source of health information for all participants, followed by television health programs. There was a general interest shown in the intelligent health assistant, with some participants commenting:

“It’s exactly what I need.” (Female, 38 years, no migrant background)

“Yes well, if I had it, I would use it.” (Male, 13 years, Turkish migrant background)

“That I really could picture well to myself, because that’s how it really is when one has questions I mean, that one can access Information quickly and informally.” (Female, 41 years, no migrant background)

“Yes, well, for health. Just looking at a medical book is enough to make me lose interest in leafing through it, because it’s so thick. And when I just imagine that I have just this thing and can simply do this, I find that great.” (Female, 41 years, no migrant background)

“…oh, maybe I would get so attached to it and ask it everything (laughs)—Should I eat this, should I not eat that? Would I be able to tolerate it (the food)?” (Female, 34 years, Turkish migrant background)

“So, I imagine that questions which concern you come, e.g., in Summer, do you feel tired and are you lethargic? Then one thinks well, actually yes and then the answer would immediately appear, somehow a picture with a strawberry salad.” (Male, 29 years, Brazilian migrant background)

“The assistant should tell one to drink something or to eat fruit, and in the evening tell you that you have taken too much sugar or fat, eaten too little fruit and drunk too little.” (Male, 40 years, Turkish migrant background)

The most marked differences observed in the needs assessment were age-related. Participants aged up to 30 years placed more worth on the IHA being interactive and wished for game elements. On the other hand, older participants preferred a more structured information platform with high quality information from dependable sources.

#### 3.2.2. Phase 2: Initial Evaluation of Prototype

Generally the participants were satisfied with the way the information and the prevention service were presented and also the health topics covered. All 13 participants rated the nutrition assistant as being very good, whereby some saw its advantages as lying mostly in weight control and/or reduction. The recipe and nutritional value functions were also reported to be very important. The participants also positively rated the fact that the nutrition and physical activity assistants were linked, with the general theme being that they would use both these functions in their everyday lives.

The main critical point raised by the participants was that there were not enough pictures and videos offered as alternatives to the information or applications. This, according to them, would make it difficult for persons with limited reading and writing abilities to fully use the application.

“I can’t write Turkish well and can only read very slowly. One doesn’t even want to read if it’s written in German. Pictures would be good—videos too.” (Female, 34 years, Turkish migrant background).

#### 3.2.3. Phase 3: Telephone Interviews

Regarding the background setting of the physical activity assistant, the few participants who took part in the telephone interviews reported that they wished for a real natural training environment, which could be changed. They also wished for the possibility to change the features of the avatar (trainer), e.g., personalize it with the face of the user, or use a comic figure.

Concerning the nutrition assistant, the participants positively rated the use of personalised data to suggest recipes or provide related information and/advice.

#### 3.2.4. Focus Group Discussion

The participants generally found all aspects of both the health information and the prevention service of the IHA easy to use and navigate. They especially positively rated the language set up and variability of the IHA, the fact that one could formulate concrete questions in the context of specific topics, and the provision of links to further information. The participants also found it useful that the IHA also considered the individual context when giving information and/or advice, e.g., regarding nutrition. The physical activity assistant was found to be the easiest to navigate. Further, the participants liked the fact that it offered the chance to do sports at home. They, however, also reported that the graphic of the function needed improving and that the trainer was not motivational enough.

The participants reported that whether or not their suggestions and recommendations for improvement would be taken into consideration when further developing the IHA would play a main role in their decision making process. Furthermore, they reported that they would only recommend the IHA to their friends, family, and acquaintances if a contact pool for users to chat with each other and share experiences were provided. There was also a general wish for information to reduce target group specific misapprehensions. That is, to focus on known misapprehensions in the Turkish community and correct the misinformation. One example cited was that, among Turkish people, the consumption of unlimited amounts of fruits and juices is generally believed to be healthy.

### 3.3. Cultural Sensitivity

Regarding the cultural sensitivity of the IHA, all participants mentioned that language was an important issue. There was consensus that the IHA should be multilingual. In particular, the so-called “first generation” Turkish migrants, regardless of their length of stay in Germany, said it would be helpful to be able to search for and read information in Turkish.

“If it is in Turkish, I understand everything.” (Female, 51 years, Turkish migrant background)

“If I don’t understand, I can switch to Turkish.” (Female, 43 years, Turkish migrant background)

“It’s good when you don’t understand something or the whole text, for example, you can read it and see it in another language.” (Male, 16 years, Turkish migrant background)

While the bilingual set up was highly appreciated, the participants felt that there was no special need for the language control function to mix the languages.

Regarding other aspects of cultural sensitivity, the participants were least satisfied with the nutrition related contents. In their opinion, the acceptance of the nutrition assistant largely depends on the selection of the recipes presented, in particular how the food tastes and the consideration of cultural values. There was general consensus that the recipes presented in the IHA were neither well selected nor sufficiently culturally sensitive.

“I mean Turkish people are—there are Turkish people who eat pork and drink alcohol. If these are included in the ingredients, one could highlight this or restrict the search.” (Female, 40 years, Turkish migrant background).

The participants found that neither the Turkish cuisine nor taste preferences of Turkish users were represented in the nutrition module. In addition, the participants also expressed a wish for advice on healthy nutrition during the fasting period: e.g., which food types are best suited for the beginning and end of fasting. While the participants rated all services offered by the IHA as being useful, only some thought they would actually use all of them in day-to-day situations.

Following the demonstration of the pregnancy support function during the second phase of the study, the participating Turkish women (*n* = 6) generally positively assessed the function and found it to be helpful, while the German women (*n* = 2) tended to be more critical in their assessment. The latter feared that the use of the function would lead to loss of personal contact to other women. They however also said that the function might be useful for those with poor command of the German language and/or not familiar with the German health system.

“That’s a nice pleasant thing—she doesn’t have to go somewhere where she will encounter difficulties. It’s comfortable—she can go in and inform herself about everything. That’s a nice thing.” (Female, 51 years old, Turkish migrant background)

“It’s something which happens often. I mean, I’ve had similar experiences very often. I’ve observed this in my surrounding quite often. For example quite often I go along as a translator because of such things, because they need information, have some questions they can’t ask because they don’t speak and understand German well. That’s why it’s necessary—it is better in Bremen, there are two Turkish gynecologists—the women can inform themselves better, but such a thing would be natural—I mean she could inform herself from her home and needn’t go all the way to the doctor for every little thing. She can ask everything there and get the necessary information.” (Female, 23 years, Turkish migrant background)

“Pregnancy needs life, I mean needs interaction, needs closeness, needs trust, the talks. I can’t even imagine using that thing in such circumstances.” (Female, 41 years, no migrant background)

“I could well imagine that it would help someone who is new here and is not familiar with the structures.” (Female, 32 years, no migrant background)

Although all participants found that the consideration of cultural aspects was important in the pregnancy support function, in contrast to Germans, Turkish participants did not think that this was of vital importance.

“But where is the difference, I ask you. Is a pregnant Turkish woman different to a pregnant German woman?” (Female, 40 years, Turkish migrant background)

However the participants could not clearly pinpoint, which cultural aspects regarding pregnancy and pregnancy support should be considered and implemented in the module on prevention.

Turkish families generally stated that they preferred getting health advice from an IHA more than from health care personnel, whereas German families tended to prefer personal contact to health care services.

“I don’t know, it is easy and—if the person gets the right answer immediately, it is easier than going to the doctor.” (Female, 34 years, Turkish migrant background)

## 4. Discussion

In this study we assessed the acceptance and usability of a newly developed “intelligent” health assistant among Turkish and German families in Bremen. We successfully used a participatory approach with families of different backgrounds in the development phases of an Internet-based health tool. Further, our study shows the importance and advantages of involving members of the intended user population when developing and designing tools, such as the IHA. Generally, all participants welcomed the idea of an IHA, as long as it would be incorporated in existing technological devices such as smartphones.

There are many studies which have investigated the use of health-related Internet offers and smartphone applications as a source of information for the general population, e.g., cancer information [18] or pain management [19]. The majority of such studies however did not involve the populations they intended to serve in the development process of the respective information [15,16]. Further, even fewer studies have reported on the use and acceptability of Internet-based, respectively smartphone-based health-related offers focusing on groups that are often disadvantaged in terms of health and access to healthcare. Among these are migrants and their descendants.

To make health services more accessible to migrants, it has been suggested that they should be provided with information about health-related topics and the health system in their own language [20], and that the issue of language barriers in service delivery be addressed [21]. Measures to overcome language or cultural barriers include the use of easily accessible and free professional translation services and the training of health workers regarding culturally-sensitive approaches [21,22]. In Germany, there are programs in which trained mediators of different ethnicities are used to facilitate access of migrants to health information and to the health system or to disseminate health-related information during German language classes for newly-arrived migrants [23]. However, these specific programs are expensive to run and need specially trained personnel and time [8].

In our study, we found that Turkish families preferred to get health advice from an IHA more than from health personnel. This preference could be associated with the language and cultural barriers mentioned before. The IHA developed and tested in our study overcame the language barriers as the language of use could be determined by the user. The interesting difference in attitudes towards the IHA’s pregnancy support function we observed between Turkish and German families is most likely linked to the issue of language and/or cultural barriers. Whereas German families were reserved about this feature, fearing a potential loss of personal contact with service providers and other pregnant women, Turkish families welcomed such a service. The difference in preference between the two groups could, thus, be due to the fact the former are not faced with the language or cultural barriers faced by the latter.

In line with the findings of a qualitative review focusing on technology-based interventions for weight loss [24], most of the participants in our study expressed the wish for a contact pool for users to chat with each other and share experiences. In the review which included 21 studies, social support was found to be one of five key components of the interventions. The other four were self-monitoring, counsellor feedback, and communication and the use of an individually tailored program [24], aspects which are also covered in the IHA developed in this study. The IHA was also personalized and the users could access information in their language of choice (German or Turkish) and adapt the information by providing their personal context, e.g., pre-existing medical condition.

Studies which have developed computer-based programs comparable to ours have reported similar findings, particularly regarding acceptability. In the LUCHAR-study in which a computer program designed for delivery in community settings to encourage healthy diet and increase physical activity among Latinos living in the United States was developed, program users received personalized feedback on nutrition, physical activity, and smoking behaviors. The authors observed a high acceptability of the intervention and significant improvements in nutrition and physical activity. However, no changes in smoking behaviors were observed [25]. In a further study in which a cardiovascular disease prevention website for Latinos was developed, focus groups discussions with community members showed that for it to be accepted and used, the website had to be (i) culturally appropriate; (ii) colorful and eye-catching, but also professional; (iii) easy to navigate with simple and easy-to-understand-information; and (iv) interactive, with immediate feedback on health [26]. All these factors were also mentioned by Turkish families in our study, especially concerning nutrition-related programs.

### Strengths and Limitations

A major strength of our study is the participatory development of the IHA and the provision of professionally-created health care information, assembled by healthcare professionals and experts in the respective fields, e.g., nutritionists and obstetricians [27,28]. The scientific validity of health-related web information, including apps targeting the general population, is of core concern, as such information carries the potential to endanger patient safety [29]. For example, in a review evaluating available smartphone applications for chronic pain, the majority of the identified 220 sites were found to include pain-related apps which not only lack evidence of healthcare professionals’ input during development, but also contained only a few evidence-based pain management features [30]. Some programs have also been developed without the incorporation of the prospective users. In our study, the IHA was developed in close cooperation with the participating families and included pre-usability evaluation (Phases 2 and 4). Participants were involved in the development process early and they could see how their suggestions were being incorporated at further stages of the study.

The fact that the interviews were conducted at places chosen by the participants helped contribute towards a relaxed and trusted atmosphere. Including Turkish and German families in the study enabled comparisons between the two groups. Our findings in this regard confirmed the existence of some differences in attitudes towards and perception of health care services. This was particularly evident during the discussions on the pregnancy support function.

To our knowledge there is no other study on the use and acceptability of Internet health offers, respectively the use of health-related apps or studies focusing on the development, usability, and acceptability of a bilingual health application in Germany.

Our findings are not generalizable, as we cannot rule out that the individuals we recruited via the community, family, and youth centers are already well-informed, respectively, have access to certain services. Their attitudes/perceptions might, thus, be different to those of persons who do not attend such institutions.

A further limitation is the small number of Turkish and German families who participated in the interviews, particularly in the second and third study phases, as well as in the focus group discussions. This was, however, a feasibility study, based on an exploratory project and, therefore, the aim was not to include large numbers of participants. During the focus group discussions, participants had a guide who supported them throughout the session, thereby making it easier for them to navigate and use the IHA. Hence we do not have any feedback on the usability of the IHA under unguided conditions. In addition, participants only used the IHA for limited periods of time during Phases 2 (up to four hours) and 4 (one-day workshop), thereby limiting our findings on usability. The use of a structured list of items to be evaluated through a Likert scale could also have improved the assessment of the acceptability and usability of the IHA. Furthermore, as the aim of the project was to develop and test a prototype, we do not yet have information on the implementation and sustainability of this approach.

## 5. Conclusions

Participants were generally open to the idea of an intelligent health assistant which is integrated in existing appliances, is user-friendly, mobile, and available everywhere via the Internet. Factors which participants felt should be taken into consideration when designing an IHA included age of user, language and, for certain functions, cultural aspects. The participants were least satisfied with the nutrition component, which they felt should include recipes and ingredients from the culture of origin, and include specific aspects of food preparation. Involving the intended users in the development of tools, such as the IHA, can help provide valuable information towards the improvement of the end product. Our study indicates that well-developed IHAs could be a viable source of health information for population groups with poor access to health services. Furthermore, the IHA would not only be easily accessible via the Internet, in the long run it could help save resources, *i.e.*, personnel, money, and time.
